# Time for health change: promoting community-based diabetes screening and prevention with video vignettes and social marketing

**DOI:** 10.1186/s12889-024-19553-z

**Published:** 2024-08-28

**Authors:** Feras Jirjees, Sanah Hasan, Ines Krass, Ward Saidawi, Mohammed Khalid Al-Juboori, Amna M. Othman, Karem H. Alzoubi, Hamzah Alzubaidi

**Affiliations:** 1 College of Pharmacy, University of Sharjah, Sharjah, United Arab Emirates; 2 College of Pharmacy and Health Sciences, Ajman University, Ajman, United Arab Emirates; 3 Center of Medical and Bio-allied Health Sciences Research, Ajman University, Ajman, United Arab Emirates; 4 School of Pharmacy, University of Sydney, Sydney, NSW Australia; 5 Research Institute for Medical and Health Sciences, University of Sharjah, Sharjah, United Arab Emirates; 6 School of Pharmaceutical Sciences, Universiti Sains Malaysia, Penang, Malaysia; 7 School of Medicine, Deakin Rural Health, Faculty of Health, Deakin University, Warrnambool, VIC Australia

**Keywords:** Health service, Pharmacist-led, Pharmacy-based, Diabetes screening, Diabetes prevention, Video vignette

## Abstract

Meaningful communication between health service users and providers is essential. However, when stakeholders are unfamiliar with new health services, innovative communication methods are necessary to engage them. The aim of the study was to create, validate, and evaluate a video-vignette to enhance stakeholders’ (physicians, pharmacists, and laypeople) engagement and understanding of an innovative pharmacy-based diabetes screening and prevention program. Also, to assess the video-vignette’s capacity to measure appetite and appeal for such preventive programs. This mixed-methods study consisted of two phases. In phase one, a video-vignette depicting the proposed screening and prevention program was developed and validated following established international guidelines (*n* = 25). The video-vignette was then evaluated by stakeholders (*n* = 99). In phase two, the video-vignette’s capacity as a communication tool was tested in focus groups and interviews to explore stakeholders’ perspectives and engagement on the proposed service (*n* = 22). Quantitative data were analyzed descriptively, while qualitative data underwent thematic analysis. In total, 146 stakeholders participated. The script was well-received, deemed credible, and realistic. Furthermore, the video-vignette received high ratings for its value, content, interest, realism, and visual and audio quality. The focus groups and interviews provided valuable insights into the design and delivery of the new service. The video-vignette compellingly portrayed the novel pharmacy-based diabetes screening and prevention service. It facilitated in-depth discussions among stakeholders and significantly enhanced their understanding and appreciation of such health services. The video-vignette also generated significant interest in pharmacy-based diabetes screening and prevention programs, serving as a powerful tool to promote enrollment in these initiatives.

**Supplementary Information:**

The online version contains supplementary material available at 10.1186/s12889-024-19553-z.

## Background

Effective health communication and education have gained increasing recognition globally as critical to health promotion, disease prevention, self-management, the design and use of new health services, and provider-patient relations [1, 2].

Recent literature demonstrates the benefits of grounding health promotion and disease prevention interventions, including diabetes screening and prevention campaigns, in health communication theories and frameworks [3–7]. Understanding the cultural aspects of target communities enhances intervention success, and social marketing proves a practical framework that increases health-related behavior acceptability among populations [8].

When healthcare providers and members of the public lack familiarity with the structure and potential value of a new health service, storytelling is an emerging innovative approach that offers a vehicle for relating the experiences of patients with long-term illnesses and bridging the knowledge gap between healthcare providers and the public [9]. It has been suggested as an effective method of communicating scientific information to nonexperts and changing people’s knowledge and attitudes about public health issues like diabetes screening and prevention programs [9, 10]. Storytelling may be usefully deployed when designing and implementing health services in new settings. For example, since screening services and disease prevention programs are not offered in community pharmacies in many Arabic-speaking countries, pharmacists, the public, and physicians may need a greater understanding of how the services are used to screen, refer, and manage at-risk individuals. To bridge this gap, storytelling may be utilized to communicate detailed information about these services to the stakeholders, helping to meaningfully elicit their needs, preferences, and feedback, and subsequently in promoting participation and engagement with these services [6].

Under the umbrella of storytelling, scripted video-vignettes have been used as research tools that depict hypothetical patient-provider interactions to analyze the effects of specific communications on patient outcomes [11]. Results of video-vignette yielded informative data on patient perceptions and evaluations of physicians’ encounters [12–19], patient preferences for consultation styles [12, 20–25], intended treatment decisions [20], self-disclosure and trust [17], compliance and decision making [18, 19], and cognitive outcomes [24, 25]. However, research using scripted video-vignettes to depict new health services that are not routinely provided is rare [11]. In pharmacy research, video-vignettes were used to introduce advanced pharmacy services to members of the public and assess their willingness to pay for such services [26, 27]. Results of studies utilizing scripted video-vignettes indicate that they are perceived as realistic [19, 23, 25, 28, 29], permit the evaluation of a wide range of communications [19, 30–32], and allow observers to immerse in the portrayed scenario [24, 25, 28], yielding informative and valid data. However, current guidelines on the methodological approaches that might be applied in developing and conducting video-vignette research are scarce [11, 33].

We aimed to contribute to the field by detailing the process of creation, validation, and evaluation of a video-vignette depicting an innovative pharmacist-led diabetes screening and prevention service in the United Arab Emirates (UAE). Moreover, our objective was to measure the video-vignette’s capacity as a tool to enhance stakeholders’ comprehension and involvement while assessing their interest and willingness to participate in the screening and prevention program.

## Methods

**Fig. 1:**
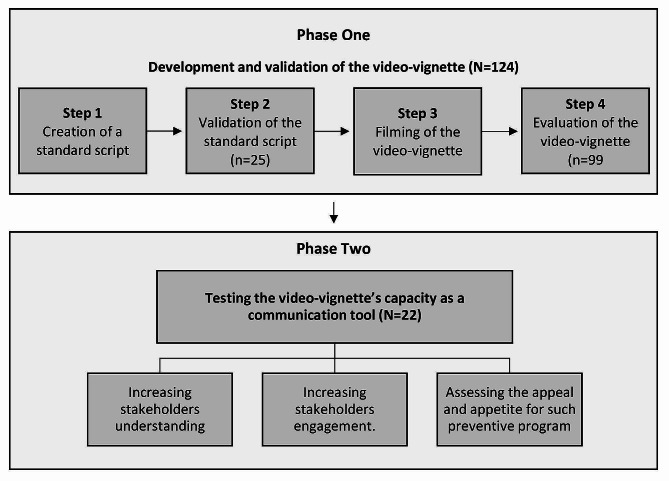
Process of creating and validating the video-vignette and testing its capacity as a communication tool

This mixed-method study was conducted in the UAE, and the primary data were collected quantitatively and qualitatively. It consisted of two phases: (i) published guidelines for the development of scripted video-vignettes were adapted, and a four-step process, described below, was employed to develop and validate the video-vignette [14], and (ii) testing video-vignette’s capacity as a communication tool to increase stakeholders’ engagement and understanding of pharmacist-led diabetes screening and prevention service. Study phases are shown in Fig. 1.

### Phase one: Development and validation of the video-vignette

#### Step 1: Creation of a standard script

A three-segment script depicted the proposed health service, following a service user through opportunistic screening, physician referral, and enrollment in a diabetes prevention program. Three experienced researchers in pharmacy health services (HA, FJ, and SH) developed the initial content and structure. The first segment portrayed a middle-aged man named Rashid visiting a community pharmacy for a vitamin supplement, where he received free diabetes screening using a point-of-care HbA1c test. The HbA1c result exceeds the cut-off value for prediabetes, and the community pharmacist counsels Rashid and refers him to a physician for further testing. The second segment describes the referral process to a physician, in which the pharmacist completes a referral letter to a physician. The client visits the physician, who confirms his diagnosis of prediabetes. The final segment describes the community pharmacy-based diabetes prevention program. The script’s internal validity was established through various sources, including publications on international diabetes screening and prevention programs, consultations with experts, and local healthcare professionals.

A professional translation service and two bilingual researchers translated the script into Arabic. The research team compared three versions of the Arabic translation and resolved minor differences in colloquial terms through discussion to better suit the target audience. This rigorous review process ensured that the script was relevant and appropriate to Arabic-speaking populations.

#### Step 2: Validation of the standard script

Purposively selected experts in health services research, physicians (specialists in internal medicine, family medicine, or endocrinology), and a convenience sample of community pharmacists and members of the public (people aged 30 years or older, with or without diabetes) evaluated the English and Arabic scripts onsite. The scripts were evaluated for internal validity (credibility of the described model of care and accuracy of medical information), external validity (realism of model of care and settings described), and language clarity. A questionnaire adapted from Van Vliet et al. was used [33], comprised of four items to assess the aspects mentioned earlier for each segment, and responses were recorded using a 3-point Likert-type scale (1 = not at all, 2 = partially, 3 = very). Open-ended items were included to allow for further comments. Feedback led to minor script modifications for improved clarity.

#### Step 3: Filming of the video-vignette

Scenes were filmed following the script, enhancing realism by using a real community pharmacy and outpatient clinic. Actual physician and pharmacist portrayed their characters and dressed accordingly. Various camera placements, movements, and field sizes were employed to show different perspectives and settings. Camera viewpoints were alternated for engagement and smooth transitions. Shots were repeated as necessary. Separate audio descriptions in English and Arabic were integrated, along with a short introduction providing context for the new service. The research team reviewed and refined the video based on feedback, ensuring its quality. A short audiovisual introduction was included at the beginning of the video to provide context for the new health service and outline the content of the video-vignette.

#### Step 4: Evaluation of the video-vignette

A purposive sample of 99 stakeholders (physicians, community pharmacists, and members of the public who are likely to use the proposed service) evaluated the English and Arabic videos. Physicians with specialties in internal medicine, family medicine, and endocrinology were recruited from various primary and secondary healthcare settings because these specialties would be more likely to receive referrals of at-risk individuals. Community pharmacists were recruited from chain and independent community pharmacies, while members of the public who were at least 30 years old and overweight or obese (i.e., representing potential users of the proposed health service) were recruited from community pharmacies. After watching the video-vignette, stakeholders completed an onsite evaluation form with two sections: one for sociodemographic information and the other with 14 statements assessing the video-vignette across three domains: (a) value and content, (b) interest and realism, and (c) visual and audio quality, which were developed based on the validated Video Engagement Scale by Visser et al. [34]. Stakeholders used a 7-point Likert scale to rate their agreement (1 = completely disagree, 7 = completely agree).

### Phase two: Testing video-vignette’s capacity as a tool to increase stakeholders’ understanding and engagement

Focus groups were conducted with community pharmacists and members of the public, while individual interviews were conducted with physicians. After watching the video-vignette, stakeholders’ perspectives on the pharmacist-delivered diabetes screening and prevention service were elicited. We created semi-structured interview guides based on the 4Ps model of social marketing, which includes product, price, place, and promotion. The interview guides explored with participants the utility of the video-vignette in explaining the proposed service and its potential as a tool to promote the engagement of these stakeholders in the service [35]. First, participants were asked to talk about their understanding of the new service and the extent to which the video-vignette helped them to ‘visualize’ how it would be offered in the community pharmacy, particularly compared to other methods of learning about the service (e.g., reading a textual description). Participants were also asked about their opinions on whether the video-vignette made the service seem more appealing and how willing they were to participate in the service after watching it (i.e., the value of the video-vignette in promoting the service). Questions relating to ‘product’ assessed participants’ perspectives about the value of the service in the UAE and their thoughts about interprofessional collaboration (asked of pharmacists and physicians only). Questions about ‘price’ centered around the various perceived direct and indirect costs of participating in the proposed service; for example, pharmacists were asked to discuss issues related to resources, time, and workload, while members of the public were asked about the time commitment and the effort needed to implement lifestyle changes. Finally, questions about ‘place’ were asked about participants’ perspectives of the pharmacy as a venue for the service. The participants in the focus groups and individual interviews were purposefully recruited and were different from the individuals who completed the quantitative evaluation of the video-vignette. The focus groups and individual interviews were conducted remotely using Zoom Meetings and were audio-recorded and transcribed verbatim.

### Data analysis

Descriptive statistical analysis was performed using IBM SPSS Statistics Version 27. Script validation data and participant characteristics were presented as frequencies and percentages, while the level of agreement with video-vignette statements was reported as means and standard deviations.

Braun and Clarke’s six-stepped thematic analysis approach was used to analyze qualitative data from focus groups and interviews [36]. Arabic transcripts were translated into English, and two authors with expertise in qualitative research independently coded and discussed the data to establish consensus on code definitions. Discrepancies were resolved through team discussion. Codes were organized into themes based on the 4Ps model of social marketing. Thematic analysis was conducted using QSR International NVivo Version 11.

## Results

### Phase one

#### Steps 1 and 2: Creation and validation of the standard script

Twenty-five participants (three physicians, 13 community pharmacists, and nine members of the public) completed the script validation questionnaire; 15 for the English script and 10 for the Arabic script. Physicians and community pharmacists were asked to evaluate all aspects of the scripts (realism, credibility, accuracy, and clarity). In contrast, the members of the public were asked to evaluate the clarity aspect of the scripts only.

Table 1 offers an overview of the participants’ responses to the three segments of the standard script. The table evaluates these segments on four aspects: realism, credibility, accuracy, and clarity.

**Table 1:** Stakeholders’ responses to the script validation questionnaire (*N* = 25)

Aspects		Segments of the standard script		
		Screening *n* (%)	Referral *n* (%)	Diabetes prevention program *n* (%)
**Realism**	Not at all realistic	0 (0.0)	1 (6.2)	1 (6.2)
	Partially realistic	9 (56.2)	2 (12.5)	7 (43.8)
	Very realistic	7 (43.9)	13 (81.2)	8 (50.0)
**Credibility**	Not at all credible	0 (0.0)	0 (0.0)	0 (0.0)
	Partially credible	4 (25.0)	5 (31.2)	7 (43.8)
	Very credible	12 (75.0)	11 (68.8)	9 (56.2)
**Accuracy**	Not at all accurate	1 (6.2)	0 (0.0)	0 (0.0)
	Partially accurate	3 (18.8)	0 (0.0)	4 (25.0)
	Very accurate	12 (75.0)	0 (0.0)	12 (75.0)
**Clarity** ^*^	Not at all clear	0 (0.0)	0 (0.0)	1 (4.0)
	Partially clear	3 (12.0)	2 (8.0)	5 (20.0)
	Very clear	22 (88.0)	23 (92.0)	19 (76.0)

^*^ Clarity was evaluated by all stakeholders (*n* = 25), while realism, credibility, and accuracy were evaluated by the physicians and pharmacists only (*n* = 16).

Looking at the realism aspect, the referral segment was viewed as very realistic by 81.2% of respondents. However, the responses were more varied in screening and in the diabetes prevention program segments with 56.2% and 43.8% of respondents finding them to be partially realistic, and 43.9% and 50% finding them to be very realistic, respectively.

The majority of the participants found the standard script to be very credible, with 75%, 68.8%, and 56.2% of the respondents reporting “very credible” in the screening, referral, and diabetes prevention segments respectively.

Similarly, 75% of the participants found both the screening and the diabetes prevention program segments to be very accurate.

Overall, while almost all participants agreed that the script segments were clear, some respondents noted that the use of complex medical terms made the script information to be only partially clear and realistic. These terms were identified in the open-ended feedback, prompting suggestions for simpler alternatives. This feedback influenced the final video content, leading to revisions that improved clarity and perceived realism without compromising the accuracy and credibility of the script.”

#### Step 3: Filming of the video-vignette

The English and Arabic video-vignettes are available at https://youtu.be/ivlsY_aJq3c and https://youtu.be/zQwA_y5Hb9s, respectively.

#### Step 4: Evaluation of the video-vignette

A total of 99 participants evaluated the video-vignette: 9 physicians, 45 community pharmacists, and 45 members of the public. Sociodemographic characteristics are summarized in Table 2. More than half of the physicians were male, and two-thirds were above 60 years old. Most had at least ten years of clinical experience. Among community pharmacists, most were under 40 years of age, two-thirds of the pharmacists held a Bachelor’s degree, and they reported various lengths of practice experience. Of the members of the public, about half were female and held a Bachelor’s degree, and approximately 1-in-4 reported being diagnosed with diabetes or prediabetes.

**Table 2:** Sociodemographic characteristics of the stakeholders who evaluated the video-vignette (*N* = 99)

Physicians (*n* = 9)			Pharmacists (*n* = 45)			Members of the public (*n* = 45)		
Characteristics		*n* (%)	Characteristics		*n* (%)	Characteristics		*n* (%)
Gender	Male	5 (55.7%)	Gender	Male	25 (55.6%)	Gender	Male	22 (48.9%)
	Female	4 (44.4%)		Female	20 (44.4%)		Female	23 (51.1%)
Age	20 to 39 years	1 (11.1%)	Age	20 to 39 years	36 (77.8%)	Age	30 to 39 years	20 (44.4%)
	40 to 59 years	2 (22.2%)		40 to 59 years	9 (20.0%)		40 to 59 years	23 (51.2%)
	60 years or more	6 (66.7%)		60 years or more	1 (2.2%)		60 years or more	2 (4.4%)
Nationality	Iraq	3 (33.3%)	Nationality	India	18 (38.3%)	Nationality	Jordan	9 (20.0%)
	Sudan	2 (22.2%)		Egypt	7 (14.9%)		UAE	5 (11.1%)
	New Zealand	2 (22.2%)		Pakistan	5 (10.6%)		Egypt	5 (11.1%)
	Others	2 (22.2%)		Others	15 (36.2%)		Others	26 (57.8%)
Medical specialty	Family medicine	4 (44.4%)	Level of education	Bachelor’s degree	30 (66.7%)	Level of education	High school	10 (22.2%)
	General practice	3 (33.3%)		Doctor of Pharmacy	9 (20.0%)		Bachelor’s degree	23 (51.1%)
	Gastroenterology	1 (11.1%)		Master’s degree	3 (6.7%)		Postgraduate degree	9 (20.0%)
	Internal Medicine	1 (11.1%)		Others	3 (6.7%)		Others	3 (6.7%)
Years in practice	< 5 years	0 (0.0%)	Years in practice	< 5 years	17 (37.8%)	Diagnosed with diabetes or prediabetes	Yes	11 (24.4%)
	5–10 years	1 (11.1%)		5–10 years	13 (28.9%)		No	32 (71.1%)
	> 10 years	8 (88.9%)		> 10 years	15 (33.3%)		Do not know	2 (4.4%)
			Type of pharmacy	Chain pharmacy	26 (57.8%)			
				Independent pharmacy	19 (42.2%)			

Table 3 shows video-vignette evaluations by physicians, pharmacists, and members of the public. The video-vignette’s value, content, interest, realism, and visual and audio quality were highly rated by all participants. They strongly agreed that the depicted diabetes screening and prevention service was easily understood, and the video-vignette provided enough information for them to fully understand the proposed health service’s value (mean scores of physicians, pharmacists, and members of the public out of 7 were 6.8, 6.4, and 6.6, respectively). There were high agreement levels among the participants that the video-vignette was engaging and interesting, and they considered that the behaviors and appearance of the pharmacist, the client, and the physician in the video-vignette were believable. Finally, participants considered the visual and audio quality of video-vignette to be appropriate.

**Table 3:** Stakeholders’ evaluation of the video-vignette (*N* = 99)

Domains and statements	Level of agreement ^*^			
	Physicians (*n* = 9) Mean (SD)	Pharmacists (*n* = 45) Mean (SD)	Members of the public (*n* = 45) Mean (SD)	Overall (*N* = 99) Mean (SD)
**Value and content**				
I was easily able to understand the health service (pharmacist-delivered diabetes screening and prevention program) in the video	6.8 (0.8)	6.4 (1.0)	6.6 (0.9)	6.5 (1.6)
The video had enough information for me to fully understand the health service	6.3 (1.1)	6.3 (1.2)	6.5 (0.9)	6.4 (1.9)
The video made me aware of the value of the health service	6.9 (0.7)	6.5 (0.9)	6.6 (0.9)	6.6 (1.5)
The video helped me to appreciate the role of the community pharmacist in providing health service	6.7 (0.9)	6.4 (1.0)	6.8 (0.5)	6.6 (1.4)
The video would have been a better format to explain the health service compared to reading a full and plain description of the health service	6.7 (0.8)	6.4 (1.1)	6.4 (1.0)	6.4 (1.7)
The video would have been a better format to explain the health service compared to reading a written vignette	6.7 (0.8)	6.4 (1.0)	6.5 (1.0)	6.5 (1.6)
**Interest and realism**				
During the viewing, I was fully concentrated on the video	6.9 (0.7)	6.2 (1.2)	6.5 (0.8)	6.4 (1.6)
The video was interesting	6.3 (1.2)	6.2 (1.3)	6.4 (1.1)	6.3 (2.1)
The client’s (Rashid) behavior and appearance in the video were believable	6.7 (0.8)	6.0 (1.3)	6.4 (0.9)	6.2 (1.8)
The pharmacist’s behavior and appearance in the video were believable	7.0 (0.6)	6.2 (1.2)	6.6 (0.7)	6.5 (1.5)
The physician’s behavior and appearance in the video were believable	7.0 (0.6)	6.3 (1.0)	6.5 (0.7)	6.5 (1.4)
The events in the video are likely to happen in real life	4.8 (2.6)	5.4 (1.8)	6.4 (1.1)	5.8 (3.3)
**Visual and audio quality**				
The visual quality of the video was appropriate	7 (0.6)	6.7 (0.6)	6.6 (0.8)	6.7 (1.2)
The voice quality of the video was appropriate	7 (0.6)	6.7 (0.6)	6.7 (0.7)	6.7 (1.1)

^*^ Stakeholders indicated their level of agreement on a scale from 1 (completely disagree) to 7 (completely agree).

### Phase two: Testing the video-vignette as a tool to increase stakeholders’ understanding, engagement. and acceptance of the new proposed service

The video-vignette was tested in two focus groups with eight community pharmacists, two focus groups with nine members of the public, and five individual interviews with physicians.

### Product

Stakeholders reflected that the video-vignette helped them to understand the proposed diabetes screening and prevention and appreciate its value, especially with the alarming rise of diabetes in the country. The diabetes screening and prevention service in UAE community pharmacies involves two phases. First, pharmacists screen individuals using a finger prick point-of-care glycated hemoglobin testing to identify those at high risk for type 2 diabetes. Second, individuals with prediabetes participate in a six-month Diabetes Prevention Program that includes education on diet, physical activity, and behavior changes, with regular follow-ups to support lifestyle modifications. They also indicated that it changed their perspective on the community pharmacy from a place for buying medicines to a potential venue to receive health services.

“*The proposed service in the video is very important because diabetes is widespread in the country and would help people to discover the risk at early stages before having diabetes” Layperson 1*.

Members of the public and pharmacists have expressed their interest in joining the diabetes screening and prevention program depicted in the video-vignette. They shared how the video-vignette helped them understand the significance of having platforms for early disease detection and prevention. After watching the video-vignette, physicians have also expressed willingness to support and participate in similar disease prevention programs.

*“After watching the video*,* I am very keen and excited to participate in such great program. I did not study all these years in pharmacy just to dispense medications and sell vitamins.” Pharmacist 3*.

*“Rashid*,* the character in the video*,* represents many people out there. I enjoyed the video*,* and I am very happy to participate in any similar program that would help me reduce my sugar level and prevent diabetes” layperson 7*.

Stakeholders reported that the video-vignette was clear, engaging, and informative. They felt they might have a lower level of attention and engagement with reading written vignettes or service descriptions compared to the video-vignette. One pharmacist also explained that she had some queries about the proposed health service and that by the end of the video-vignette, she understood what the proposed service was about, how it worked, and its value.

*“I believe the video is very descriptive and explains the whole service well. If you were to hand me a piece of paper describing Rashid’s story*,* I would most likely not go through the whole thing. I guess the visual method is more appealing.” Pharmacist 2*.

### Price

Members of the public appreciated that the screening and diabetes prevention service would be offered free of charge as part of a funded research project. Some also indicated that they would be willing to pay to receive screening and structured diabetes prevention services in the future.

*“We are very convinced to participate in the service*,* even if it has some costs” Layperson 5*.

Members of the public expressed some concern about the time commitment involved in a long-term prevention program. When asked about possible mechanisms to increase engagement and completion of the program, they reported that well-trained pharmacists are essential to provide motivation, support, and continuous follow-up. Some also suggested that a smartphone application that sends daily reminders would help maintain engagement.

*“Having a mobile application would be useful to send notifications of what to do daily to motivate the patient. The pharmacist must be well trained and able to communicate and follow up with the participants.” Layperson 4*.

### Place

After watching the video-vignette, stakeholders reported that the community pharmacy is an excellent venue for delivering the proposed health services. There was general agreement that the community pharmacy is one of the most suitable and accessible places to deliver such health services.

*“People feel safe in community pharmacies. It is a healthcare setting that is easy to reach and access. It is very suitable.” Layperson 1*.

Several doctors reported how the video-vignette altered their perception of community pharmacies as places that could provide health services instead of just being outlets for selling medicines and products.

*“Watching this video made me think about how providing such diabetes screening or any similar health services would improve the perceptions about community pharmacies as more than just places to sell medicines and products” Physician 2*.

### Promotion

Members of the public suggested that a shortened version of the video-vignette could be used as a promotional tool for the proposed health service to recruit participants and that it could be displayed in the community pharmacy and on social media. They recommended removing technical details such as the height, weight, and waist circumference measurements and filling out the referral form while emphasizing the appealing features such as free, convenient testing and the benefits of the diabetes prevention service.

*“The focus needs to be more on the eye-catching information*,* such as it’s a free test*,* you do not need to be fasting to do the test*,* expected outcomes*,* and description of the prevention service.” Layperson 6*.

Members of the public also reported that the video-vignette could be used to raise awareness about diabetes screening and prevention in general and promote healthy behaviors in the community. Within this context, one participant mentioned that before watching the video-vignette, he was unaware that diabetes could be prevented.

*“The video was very helpful and informative. Before today*,* I did not know that diabetes could be prevented! Such a service would be amazing.” Layperson 2*.

## Discussion

A new communication tool was created in the form of a video-vignette that showcased a pharmacist-led diabetes screening and prevention service that is not yet a part of routine pharmacist care. The tool’s effectiveness in enhancing stakeholders’ comprehension, engagement, and eliciting their viewpoints on the suggested novel diabetes prevention program was evaluated. Stakeholders reported that the video-vignette was clear and engaging, allowing them to understand the proposed service easily. This understanding enabled a rich and informed discussion with and among the stakeholders during focus groups and individual interviews on their perspectives and preferences regarding the proposed service and yielded valuable insight into service design and delivery.

Video-vignettes have been used in a variety of healthcare and social science research fields. Most predominantly, they were used in patient-provider communication research to study the effects of specific communications on patient perceptions and preferences regarding the depicted consultation. In line with the findings of this study, video-vignettes were perceived as realistic and provided an immersive experience to the participants [11]. Participants also agreed that the video-vignette was the best format to explain the health service and that they would have had a different level of attention and engagement compared to other methods such as written vignettes. This concurs with the findings of Barter and Renold, who also reported that their participants considered the video vignette to be more interesting and gripping than the text [37]. Arif et al. also reported that watching a video-vignette was more beneficial for students than reading a patient case [38].

Video-vignettes have been used in two studies examining stakeholders’ attitudes around services that were not considered routine care. These explored general practitioners’ attitudes towards video consultations to expand healthcare delivery to patients living in remote areas, introduce members of the public to quality-enhanced pharmacy services, and assess their willingness to pay for such services [26, 27]. The authors of the studies did not provide any methodological insight on how to use video-vignettes, apart from explaining why they chose to use them. Similar to this study, the video-vignette in the pharmacy services study increased the understanding and appreciation of members of the public of the quality-enhanced pharmacy service [27].

In Table 3 the item “The events in the video are likely to happen in real life” was rated lower than other items by physicians and pharmacists. This rating can be attributed to the current healthcare landscape in the UAE, where diabetes screening and prevention services are not yet commonly provided through community pharmacies. As a result, healthcare providers in the country are unfamiliar with the operational aspects, delivery mechanisms, and benefits of such health services.

The lack of familiarity with the integration of these services into community pharmacies likely influenced the respondents’ perception of the video’s realism. Without firsthand experience or established examples of these services in practice, both physicians and pharmacists may find it challenging to envision the scenarios depicted in the video as plausible or reflective of their professional environment.

This unfamiliarity and the novelty of implementing diabetes prevention programs within community pharmacies could explain the lower rating. Addressing this gap in knowledge and experience through training and pilot programs could enhance the perceived feasibility of these services and align healthcare providers’ expectations with the potential reality of integrated community pharmacy-based health services.

Furthermore, in the study with general practitioners, the video-vignette enabled them to anticipate the potential disadvantages of video consultations for certain patients, incorporating their prior experience with patient care [26].

As demonstrated in this study and prior literature, within a short period, video-vignettes allow the communication of a reasonably large amount of information to participants while maintaining their engagement and interest and can provide an immersive experience that enables researchers to elicit participants’ preferences and perceptions about familiar or unfamiliar situations. Additionally, as noted by participants, an adapted version of the video-vignette could be used to raise awareness about the preventable nature of diabetes and the diabetes screening and prevention service and to promote engagement in the service by displaying the video in healthcare settings or on social media. Such a promotional video need only contain the essential components of the service (for example, that diabetes can be prevented and that pharmacists can help) and highlight its attractive features (for example, that the service is provided at no cost to participants and its convenience). Of note, social cognitive theory constructs such as observational learning and role-modeling can augment the effectiveness of these promotional messages by showing the positive outcomes of the video-vignette character’s enrollment in the diabetes prevention program [6]. These potential applications of the video-vignette may enhance participants’ enrolment in the screening and prevention service, and help investigators avoid some of the additional time and financial expense of filming promotional material.

When deciding whether to use the video-vignette method, it is important to consider specific challenges that may be presented. The multi-phase process for developing valid and rigorous video-vignettes can be time-consuming and requires financial resources and access to persons with the necessary expertise and equipment for filming and editing.

### Implications for practice

For Arabic-speaking countries and Western countries with Arabic-speaking immigrant populations, where disease prevention may not be a part of the culture, a well-crafted video-vignette has the potential to significantly enhance stakeholders’ understanding and appreciation of innovative screening and prevention services that are not yet part of the standard healthcare practices. It can also generate significant interest in such programs, serving as a powerful tool to promote enrollment in these much-needed initiatives.

### Limitations and strengths

This study has two major strengths: the adaptation of an evidence-based video-vignette development guideline and a robust multi-step validation process involving relevant experts and stakeholder groups ensuring the validity of the video-vignette.

Some limitations must be considered. The video-vignette could not portray multiple diabetes prevention sessions to emphasize frequent contact in the service. In addition, pharmacist remuneration could not be mentioned. However, there are constraints on the amount of information that can be contained in a video-vignette to keep it reasonably short, simple, and feasible to film.

Another limitation was the use of a 3-point Likert scale to evaluate the video-vignette script. While this scale has been validated and offers a simple way to measure participant responses, it may not be as precise as scales with more points, like a 5-point or 7-point Likert scale. By using a 3-point scale, we may have made it harder to detect subtle differences in perceptions and attitudes. In future studies, it would be beneficial to use a more detailed scale to capture a wider range of participant feedback.

Another limitation is the lack of detailed information about the cost of the service being evaluated. The absence of cost data may have also impacted participant engagement and responses, as financial considerations often play a key role in decision-making processes. Future research should include comprehensive cost analyses to help stakeholders make more informed decisions.

### Electronic supplementary material

Below is the link to the electronic supplementary material.

Supplementary Material 1

## Data Availability

The authors confirm that the datasets used and/or analysed during the current study are available from the corresponding author on reasonable request.
