# Supplementary material for: Time for health change: promoting community-based diabetes screening and prevention with video vignettes and social marketing
Source: BMC Public Health. 2024 Aug 28;24:2340. doi: 10.1186/s12889-024-19553-z (PMC11360882; doi:10.1186/s12889-024-19553-z)
Supplement: Supplementary file 1 — Supplementary Material 1 [file 12889_2024_19553_MOESM1_ESM.docx]

**Appendix 1: Script Validation Questionnaire**

*Note:* Laypeople evaluated the Clarity aspect of the script, however physicians and pharmacists evaluated all the aspects of the script.

Reflecting on the script, please answer the following questions on script realism, credibility, accuracy, and clarity.

1. **Script on community pharmacy-based diabetes screening**
   1. How realistic is the script in depicting diabetes screening as it may be delivered at the community pharmacy in the UAE?
      1. Not at all realistic
      2. Partially realistic
      3. Very realistic
   2. What do you think about the credibility of the community pharmacy-based diabetes screening program depicted in the script?
      1. Not at all credible
      2. Partially credible
      3. Very credible
   3. How would you rate the accuracy of the medical information on the community pharmacy-based diabetes screening program depicted in the script?
      1. Not at all accurate
      2. Partially accurate
      3. Very accurate
   4. How would you rate the clarity of the script in describing a diabetes screening program at the community pharmacy to those who are unfamiliar with such a service?
      1. Not at all clear
      2. Partially clear
      3. Very clear
   5. Please provide any comments to improve this segment's realism, credibility, accuracy, clarity, and structure.

|  |
| --- |

1. **Script on referral to a physician**
   1. How realistic is the script in depicting the process of referral to the physician as it may occur at the community pharmacy in the UAE?
      1. Not at all realistic
      2. Partially realistic
      3. Very realistic
   2. What do you think about the credibility of the process of referral to the physician from the community pharmacy depicted in the script?
      1. Not at all credible
      2. Partially credible
      3. Very credible
   3. How would you rate the clarity of the script in describing the process of referral to the physician from the community pharmacy to those who are unfamiliar with such a service?
      1. Not at all clear
      2. Partially clear
      3. Very clear
   4. Please provide any comments to improve this segment's realism, credibility, accuracy, clarity, and structure.

|  |
| --- |

1. **Script on community pharmacy-based diabetes prevention program**
   1. How realistic is the script in depicting a community pharmacy-based diabetes prevention program in the UAE?
      1. Not at all realistic
      2. Partially realistic
      3. Very realistic
   2. What do you think about the credibility of the community pharmacy-based diabetes prevention program depicted in the script?
      1. Not at all credible
      2. Partially credible
      3. Very credible
   3. How would you rate the accuracy of the medical information on the community pharmacy-based diabetes prevention program depicted in the script?
      1. Not at all accurate
      2. Partially accurate
      3. Very accurate
   4. How would you rate the clarity of the script in describing the diabetes prevention program at the community pharmacy to those who are unfamiliar with such a service?
      1. Not at all clear
      2. Partially clear
      3. Very clear
   5. Please provide any comments to improve this segment's realism, credibility, accuracy, clarity, and structure.

|  |
| --- |

**Appendix 2: Video Evaluation Form**

| *Please indicate how much you agree with the following statement. You can place an 'X' in the box that best represents your opinion* | | | | | | | | |
| --- | --- | --- | --- | --- | --- | --- | --- | --- |
| **No** | **Domains and Statements** | **Completely Disagree** |  | | | | | **Completely Agree** |
|  |  | **1** | **2** | **3** | **4** | **5** | **6** | **7** |
| **Value and Content** | | | | | | | | |
| 1 | I was easily able to understand the health service (pharmacist-delivered diabetes screening and prevention program) in the video |  |  |  |  |  |  |  |
| 2 | The video had enough information for me to fully understand the health service |  |  |  |  |  |  |  |
| 3 | The video made me aware of the value of the health service |  |  |  |  |  |  |  |
| 4 | The video helped me to appreciate the role of the community pharmacist in providing health service |  |  |  |  |  |  |  |
| 5 | The video would have been a better format to explain the health service compared to reading a full and plain description of the health service |  |  |  |  |  |  |  |
| 6 | The video would have been a better format to explain the health service compared to reading a written vignette |  |  |  |  |  |  |  |
| **Interest and Realism** | | | | | | | | |
| 7 | During the viewing, I was fully concentrated on the video |  |  |  |  |  |  |  |
| 8 | The video was interesting |  |  |  |  |  |  |  |
| 9 | The client’s (Rashid) behavior and appearance in the video were believable |  |  |  |  |  |  |  |
| 10 | The pharmacist’s behavior and appearance in the video were believable |  |  |  |  |  |  |  |
| 11 | The physician’s behavior and appearance in the video were believable |  |  |  |  |  |  |  |
| 12 | The events in the video are likely to happen in real life |  |  |  |  |  |  |  |
| **Visual and Audio Quality** | | | | | | | | |
| 13 | The visual quality of the video was appropriate |  |  |  |  |  |  |  |
| 14 | The voice quality of the video was appropriate |  |  |  |  |  |  |  |
| 15 | Please provide any other comments you may have on the video | | | | | | | |

**Appendix 3: Interview Guide (Physicians’ Version)**

| **Domains** | **Questions** |
| --- | --- |
| **Product** | 1. After watching the video, what are your thoughts about this service being offered in the community pharmacy to people who are at risk of developing diabetes?   ***Prompts:***   - Importance/benefits of this service for people in the UAE   1. Opportunity for community pharmacists to assist clients to reduce their risk of developing diabetes? - Acceptability - Willingness to participate - Likability - Concerns/issues with providing this service in a community pharmacy  1. **In the video**, the health service (diabetes screening and prevention program) involved a collaboration between the community pharmacist and the physician. What do you think about this collaboration?   ***Prompts:***   - Benefits to clients - Reduce workload - Conflict with physician’s roles - Willingness to collaborate |
| **Price** | 1. After watching the video, what would take from you to take part in a health service like this?   ***Prompts:***   - Busyness and time - Inconvenience - Others |
| **Place** | 1. After watching the video, the following questions are about where the diabetes screening and prevention program is offered: 2. What are your thoughts about community pharmacy as a venue to provide the screening? 3. What are your thoughts about receiving referrals from the pharmacist to confirm the prediabetes diagnosis? 4. How would you then feel about referring the prediabetic clients back to the community pharmacy for a prevention program? 5. How suitable is the community pharmacy as a venue to provide the prevention program? |
| **Promotion** | 1. How helpful **was the video** in making you understand the diabetes screening and prevention program? 2. In your opinion, which of the following would explain diabetes screening and prevention program in the community pharmacy better? For example, watching the video? Reading Rashid’s story instead of watching it? Reading brochures? Or listening to advertisements? 3. How successful **was the video** in convincing you to engage in a diabetes screening and prevention program like this?   ***Prompts:***   - Very convinced, partially convinced, not at all convinced - Why? |
| **Conclusion** | 1. Do you have any comments or remarks you like to raise? |

**Appendix 4: Interview Guide (Pharmacists’ Version)**

| **Domains** | **Questions** |
| --- | --- |
| **Product** | 1. After watching the video, what are your thoughts about this service being offered in the community pharmacy to people who are at risk of developing diabetes?   ***Prompts:***   - Importance/benefits of this service for people in the UAE   1. Opportunity for community pharmacists to assist clients to reduce their risk of developing diabetes? - Acceptability - Willingness to participate - Likability - Concerns/issues with providing this service in a community pharmacy  1. **In the video**, the health service (diabetes screening and prevention program) involved a collaboration between the community pharmacist and the physician. What do you think about this collaboration?   ***Prompts:***   - Benefits to clients - Reduce workload - Conflict with physician’s roles - Willingness to collaborate |
| **Price** | 1. After watching the video, what would take from you to engage in providing these services, and what issues come to mind?   ***Prompts:***   - Time - Being paid - Commitment - Inconvenience - Effort (learning and delivering the services beyond dispensing) - Personal discomforts (providing information, physicians attitude) |
| **Place** | 1. After watching the video, what are your thoughts about community pharmacy as a venue to provide diabetes screening?   ***Prompts:***   - Infrastructure - Privacy - Support from the pharmacy management/colleagues delivers the health services  1. After watching the video, how suitable is the community pharmacy as a venue to provide the diabetes prevention program?   ***Prompts:***   - Infrastructure - Privacy - Support from the pharmacy management/colleagues delivers the health services |
| **Promotion** | 1. How helpful **was the video** in making you understand the diabetes screening and prevention program? 2. In your opinion, which of the following would explain diabetes screening and prevention program in the community pharmacy better? For example, watching the video? Reading Rashid’s story instead of watching it? Reading brochures? Or listening to advertisements? 3. After watching the video, how convinced were you in providing a diabetes screening and prevention program like this?   ***Prompts:***   - Very much convinced, moderately convinced, not at all - Why?  1. How do you think the video could be improved to promote the diabetes screening and prevention program?   ***Prompts:***   - Scenario - Attractiveness - Understandability and clarity |
| **Conclusion** | 1. Do you have any comments/issues you like to raise? |

**Appendix 5: Interview Guide (Laypeople’s Version)**

| **Domains** | **Questions** |
| --- | --- |
| **Product** | 1. After watching the video, what are your thoughts about this service being offered in the community pharmacy to people who are at risk of developing diabetes?   ***Prompts:***   - Importance/benefits of this service for people in the UAE   - Opportunity for community pharmacists to assist clients to reduce their risk of developing diabetes? - Acceptability - Willingness to participate - Likability - Concerns/issues with providing this service in a community pharmacy |
| **Price** | 1. After watching the video, what would take from you to engage in a health service like this?   ***Prompts:***   - Time (traveling time, time off from work, extra time for exercise, etc.) - Cost (service cost, physician’s cost, traveling cost, etc.) - Inconvenience (fingerstick, changes in diet, some food restrictions, going to different healthcare professionals, etc.) - Effort (changing the behaviors, energy in exercising, etc.) - Commitment/adherence to the diabetes prevention program |
| **Place** | 1. After watching the video, the following questions are about where the diabetes screening and prevention program is offered: 2. What are your thoughts about community pharmacy as a venue to provide diabetes screening? 3. What are your thoughts about community pharmacy as a venue to provide the diabetes prevention program?   ***Prompts:***   - Pharmacist approachability and availability - Pharmacy accessibility - Privacy and confidentiality - Opening hours - Waiting times - Lack of appointments  1. What are your thoughts about being referred by the pharmacist to a physician for diagnosis? 2. How would you then feel about being referred back to the community pharmacy for a diabetes prevention program? |
| **Promotion** | 1. Do you think the video was helpful in making you understand the diabetes screening and prevention program? Please elaborate 2. In your opinion, which of the following would explain diabetes screening and prevention program in the community pharmacy better? For example, watching the video? Reading Rashid’s story instead of watching it? Reading brochures? Or listening to advertisements? 3. How successful **was the video** in convincing you to engage in a diabetes screening and prevention program like this?   ***Prompts:***   - Very convinced, partially convinced, not at all convinced - Why?  1. How do you think the video could be improved to better promote the diabetes screening and prevention program?   ***Prompts:***   - Scenario - Attractiveness - Understandability and clarity |
| **Conclusion** | 1. Do you have any comments or remarks you like to raise? |
